# Supplementary material for: Switching the spin cycloid in BiFeO3 with an electric field
Source: Nat Commun. 2024 Apr 4;15:2903. doi: 10.1038/s41467-024-47232-5 (PMC10995181; doi:10.1038/s41467-024-47232-5)
Supplement: Supplementary file 1 — Supplementary Information [file 41467_2024_47232_MOESM1_ESM.pdf]

Supplementary Data for **Switching the spin cycloid in $BiFeO_3$ with an electric field**

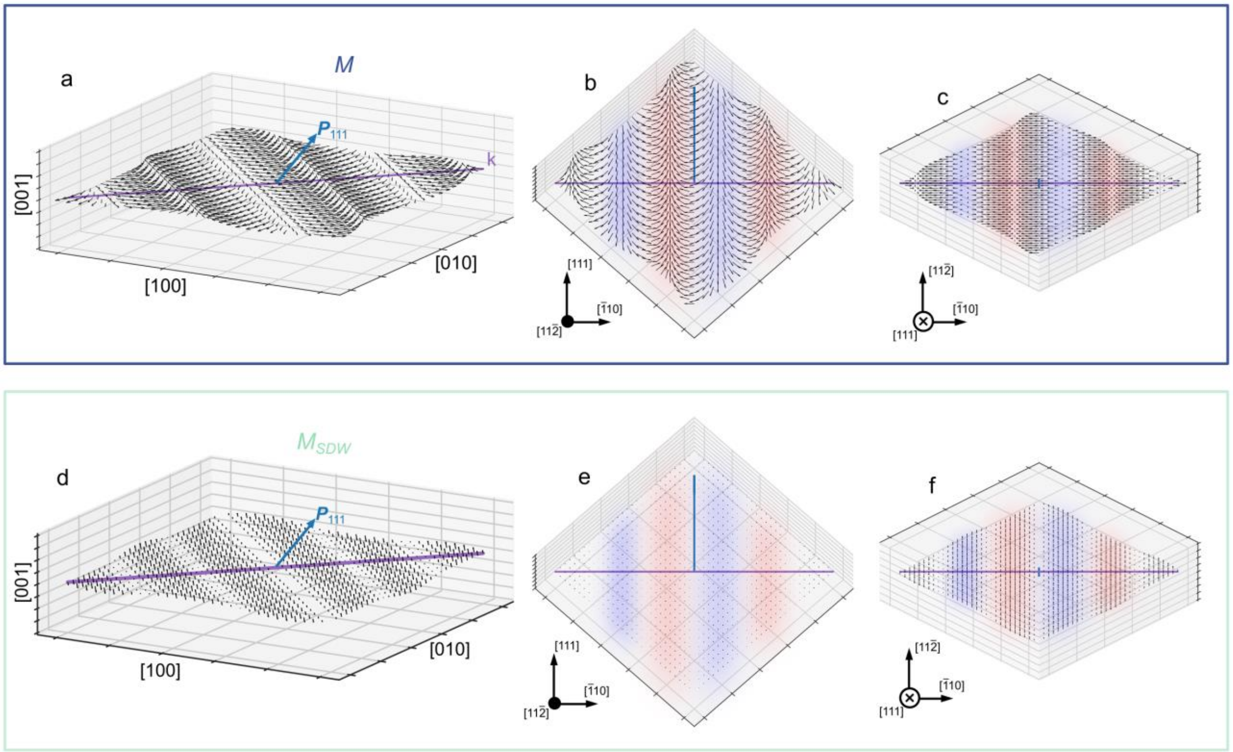

Supp. Figure S1 | 3D representation of the spin cycloid. Vector plots of solutions to **Eqs. 1** and **2**, dictating empirical descriptions of M (**a-c**) and M_{SDW} (**d-f**). Views along $[11\bar{2}]$ and $[111]$ (⊥ and ∥ to $\boldsymbol{P}$) are shown, illustrating that M rotates in the $(11\bar{2})$, defined by $\boldsymbol{P}$ and $\boldsymbol{k}$, and M_{SDW} exists in the (111) along the $[11\bar{2}]$. The red and blue shading map to the images measured via NV magnetometry. Previous works[1,2] propose that the signal observed in NV magnetometry is due primarily or completely to the component of M_{SDW}, shown here in the $[11\bar{2}]$ direction (**f**). As described in the text, M_{SDW} is largest when the Fe spins point along $[\bar{1}10]$; as M is always perpendicular to the axis of the Fe spins, M_{SDW} is largest when M points along $[111]$, shown in c and f.

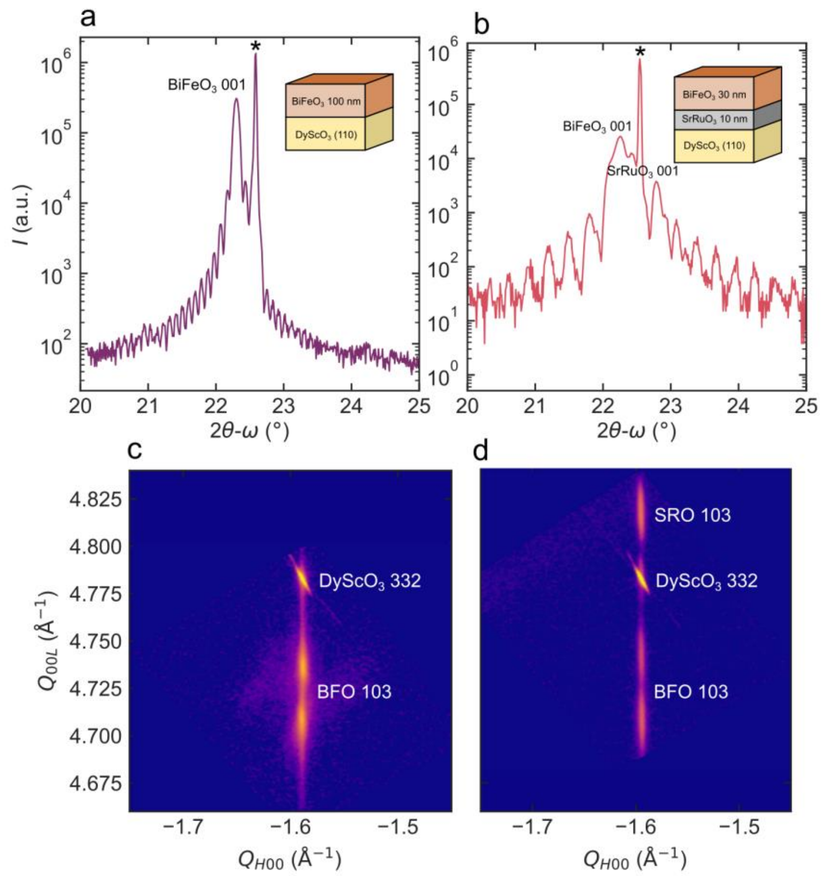

Supp. Figure S2 | XRD of $BiFeO_3$ films. Xray diffraction line scans of BFO thin films deposited directly on the substrate, **a**, and on a conducting $SrRuO_3$ back electrode, **b**, used in the experiments here. Pendellosung fringes confirm the quality and expected thickness of films. **c,d** Reciprocal space maps of the same films as a,b about the $103_{PC}/332_{O}$ diffraction peaks. Q_{hkl} is labeled with respect to pseudocubic symmetry. In both cases, the films are epitaxially strained to the in-plane pseudocubic lattice constants of the $DyScO_3$ substrate, $a \cong b = 3.952$ Å. The splitting of the BFO 103 peaks in Q_{00L} is due to the two rhombohedral variants from the two different ferroelectric domains[3]. Ferroelectric and magnetic domain structures in both samples, 100 nm and 30 nm, are approximately the same in the as-grown state, confirming their comparability.

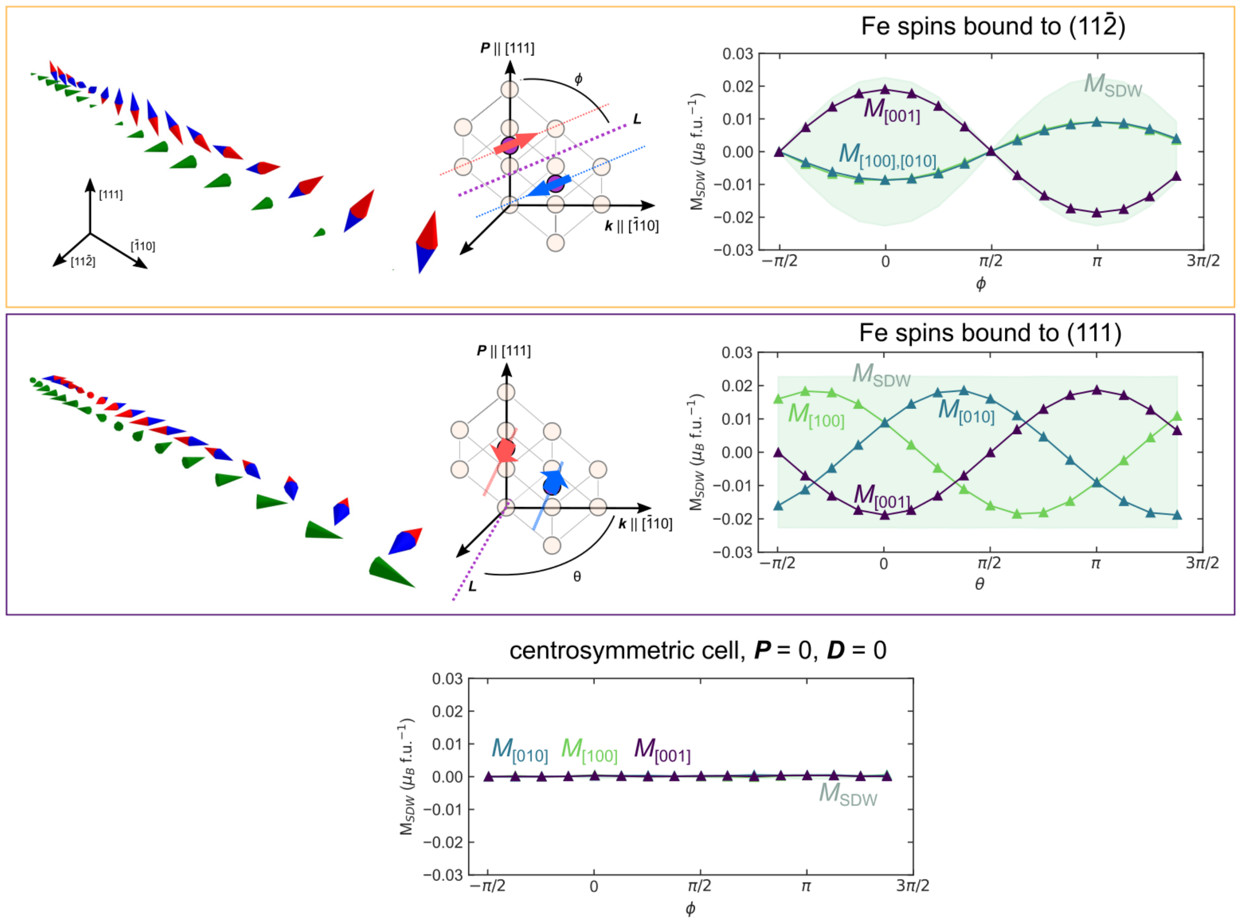

Supp. Figure S3 | Modulation of M_{SDW} due to the cycloid. a In the discretized DFT calculations, when the Fe spins are rotated in the $(11\bar{2})$, we observe the emergence of M_{SDW} in the $[11\bar{2}]$ direction due to the interaction with the DMI caused by the antiferrodistortive rotations of the FeO_6 octahedra, pointing along the direction of $\boldsymbol{P}$. From symmetry, $H_{ij}{}^{DM} = \boldsymbol{D_{ij}} \cdot (\boldsymbol{S_i} \times \boldsymbol{S_j})$ which, because $\boldsymbol{S_i}$ and $\boldsymbol{S_j}$ are bound to the $(11\bar{2})$ plane and $\boldsymbol{D_{ij}} \parallel \boldsymbol{P}$, the energy is minimized when $\boldsymbol{S_{i,j}}$ || $[\bar{1}10] \perp \boldsymbol{P}$, thus $S_{i,j}$ cants in the $[11\bar{2}]$ direction. **b** We observe that when we bind the spins to other crystallographic planes, here (111), this interaction does not form a spin density wave and instead the resultant M forms a cycloid with the same periodicity as the Fe spins. This unmodulated moment is thus compensated and would not be seen with NV microscopy[2], implying that the case in a must be approximately true in our samples. **c** As a further control, the same calculation is performed with an imposed centrosymmetry on the BFO unit cell. In this case, there is no canted moment from the simulations, confirming the mechanism of formation from $\boldsymbol{D_{ij}}$ arising due to the ferroelectric polarization.

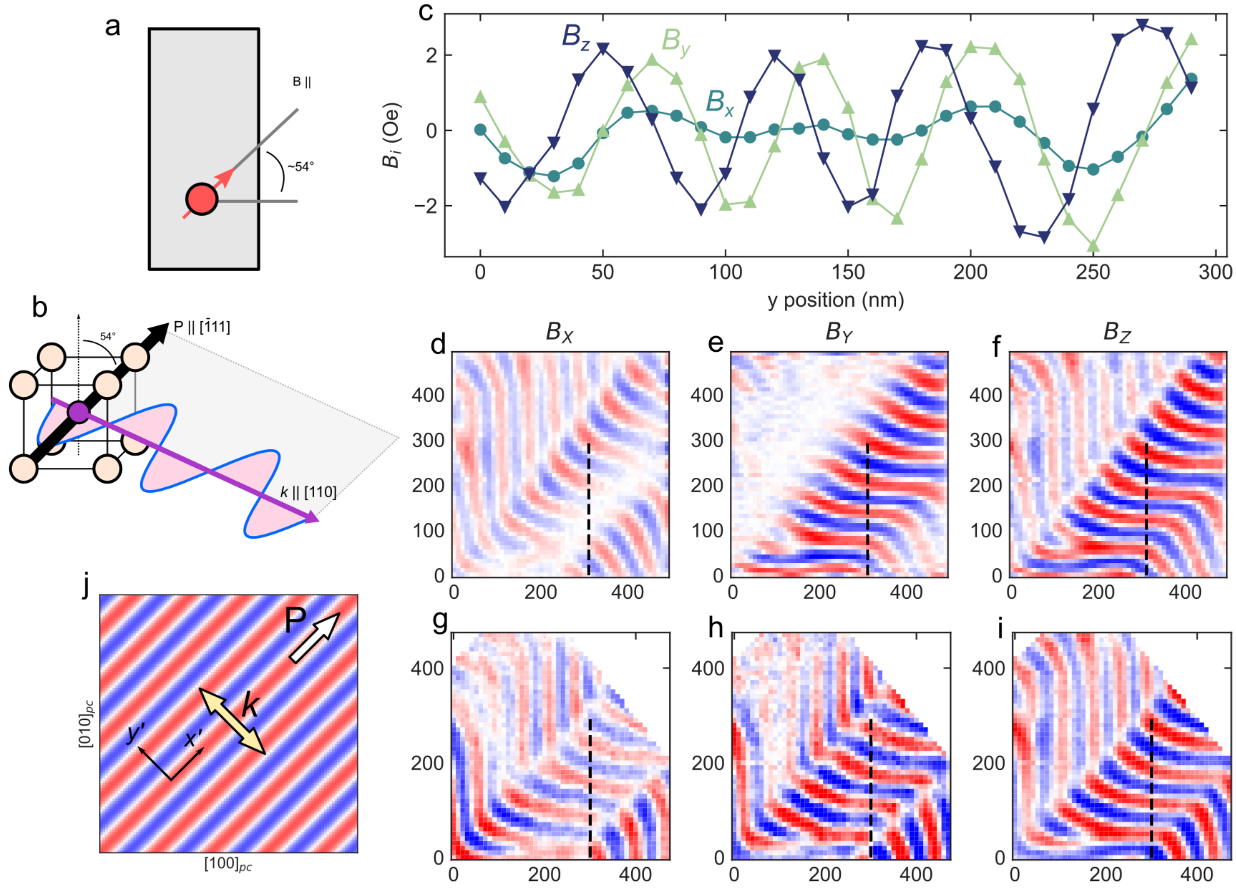

Supp. Figure S4 | Components of B reconstructed from NV data. a Schematic of the diamond tip used in NV microscopy, where the axis of the NV center points along the [111] direction of the diamond, approximately 54° from the surface (**b,j**). Using the analysis outlined in [14], the measured NV data (**c**) can be used to reconstruct the *x*, *y*, and *z* components of *B* above the sample surface (**d,e,f**). Because the components of *B* are linearly dependent in Fourier space, they can be reconstructed from a single measurement of *B* ‖ to the axis of the NV sensor. Axes are in nm. While this can be compared to a vector map of *B* reconstructed from three measurements at the same location of the sample (**g,h,i**), significant sources of error in the vector reconstruction such as uncertainties in the angle and location, as well as the fact that vector reconstruction takes three scans, make the analytical reconstruction preferable. From [2], the stray field from the spin cycloid in BFO on DSO can be expressed as:

$$B_x = A\sin\big(k(x-y)\big) \qquad (S1)$$

$$B_y = -A\sin\big(k(x-y)\big) \qquad (S2)$$

$$B_z = \sqrt{2}A\cos\big(k(x-y)\big) \qquad (S3)$$

With the cycloid propagation vector $\vec{k}$ along [-110] in the lab frame when oriented with x and y parallel to the [100] and [010] pseudocubic crystallographic axes. If the reference frame is rotated 45°, such that $\vec{k} \parallel y'$ and $\vec{k} \perp x'$, where y' and x' are along [-110] and [110] respectively (illustrated in **j**), the expression for B_i rotates such that:

$$B_{x'} = \frac{1}{\sqrt{2}}\left(B_x + B_y\right) = 0 \tag{S4}$$

$$B_{y'} = \frac{1}{\sqrt{2}}\left(B_x - B_y\right) = \sqrt{2}A\sin\big(k(x-y)\big) = \sqrt{2}A\sin(ky') \tag{S5}$$

Here, we see that only $B_{y'}$, the field along the propagation vector $\vec{k}$, should be measurable, which is reflected in this reconstruction.

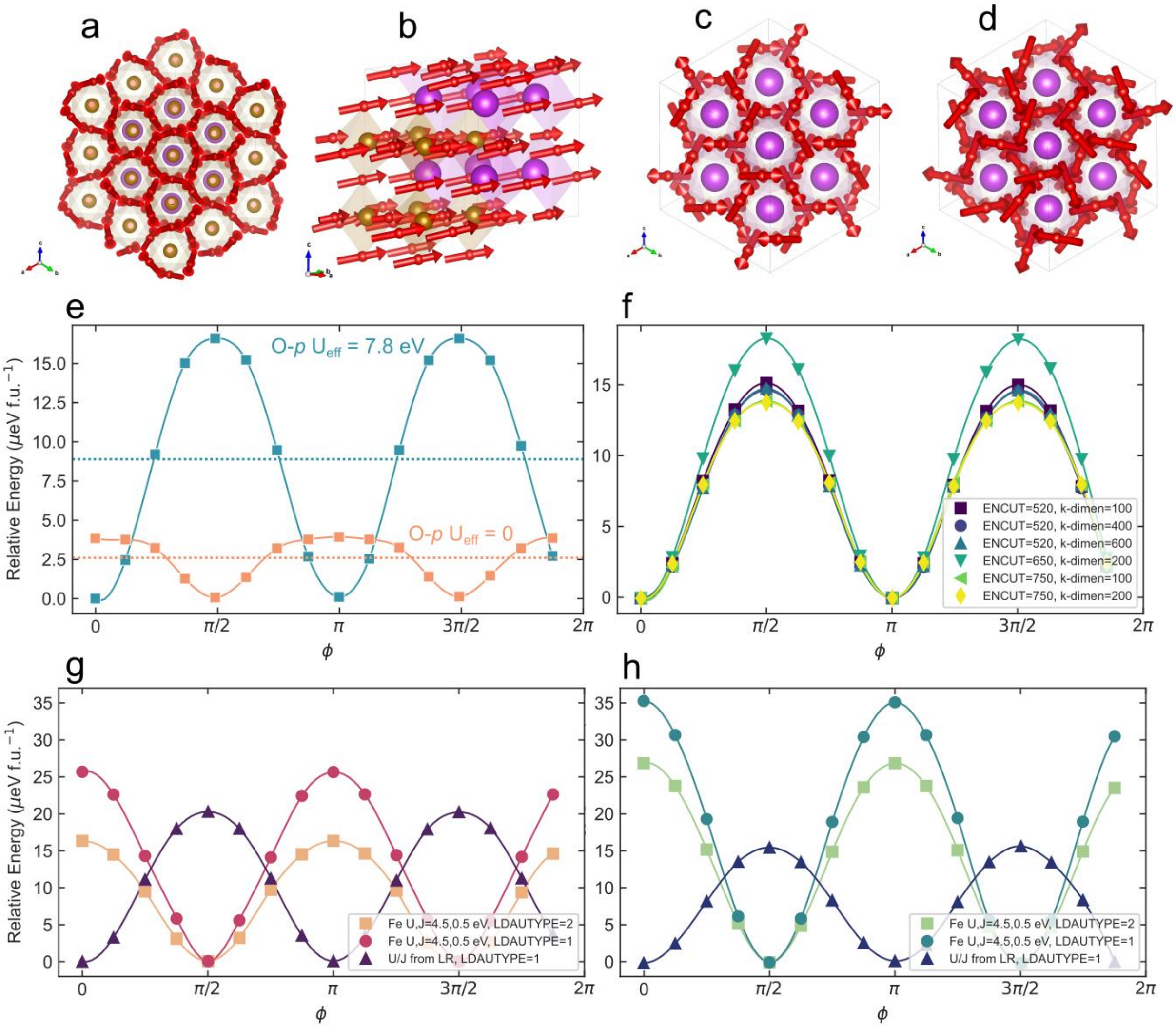

Supp. Figure S5 | DFT methodology. a-d Visualized distortions induced from various origins, as well as energy path comparisons over various DFT parameters. To provide a reference chirality of parent AFD distortions, **a,** arrows indicating the displacements of the cubic structure relative to the distorted structure, as reported in Ref. [4]. The relaxed relative distortions under strain is visualized in **b**. Additionally, **c** shows the reduction in antiferrodistortive (AFD) rotations due to the addition of Hund J applied to Fe-d, and **d**, the further reduction in AFD magnitude due to Hubbard corrections to the Bi-p and O-p manifolds. **e** Energies versus rotation angle for $\boldsymbol{k} \parallel [\bar{1}10]$, with and without U_{eff} applied to the oxygen 2p states. The input structure itself is relaxed with Fe-d: U,J = 5.2,0.4 eV and O-p: U,J = 9.7,1.9 eV Hubbard corrections. **f** Anisotropy energy for different energy cutoffs (ENCUT) and k-point densities demonstrating that we have performed a proper

convergence test versus the resolution of DFT calculations. **g,h** Energy versus rotation angle for different on-site corrections, compared for two different relaxed structures. The linear response Hubbard corrections correspond to Fe-*d*: U,J = 5.2,0.4 eV, Bi-s: U,J = 0.8,0.8 eV, and O-*p*: U,J = 9.7,1.9 eV. The structure in **g** is relaxed with these Hubbard parameters, whereas **h** is relaxed with U_{eff} = 4.0 eV applied to Fe-*d* sites.

In the unstrained calculation, the on-site Hubbard U and Hund J parameters on O-*p*, in addition to Fe-*d*, are primarily responsible for the minimum in the anisotropy energy along $[111]$, instead of the intersections with the (111) as previously reported[5]. For the U/J parameters listed above, we observe that using U/J applied to Fe-*d* states alone, the $[111]$ corresponds to a local maximum in energy. Conversely, $[111]$ is a local minimum if we apply on-site corrections to the O-*p* manifold. This result appears to be agnostic to the flavor of on-site correction used, i.e. DFT+U+J [6] versus DFT+U_{eff} [7] with $U_{\mathrm{eff}} = U - J$. This use of O-*p* Hubbard corrections could help to explain the differences between local minima on the MCAE energy landscape compared to previous DFT studies.

Additionally, when comparing DFT+U+J to DFT+U_{eff}, both an effective reduction of antiferrodistortive rotations of oxygen polyhedra, as well as a reduction of ferroelectric distortions of Fe atoms are observed. Conventionally, the inclusion of the Liechtenstein DFT+U+J formalism is known to amplify oxygen polyhedral distortions[8,9] compared to the Dudarev DFT+U_{eff} counterpart. This can be explained on the basis that, in order to motivate U_{eff}, a spherical symmetry of the Coulomb exchange integrals is assumed[10] which is rarely the most accurate assumption, especially for transition metal oxide systems with symmetry broken by crystal field splitting[8,9]. In this case, however, the possible conflict between SOC and the Hund J antisymmetric intra-orbital exchange is also considered, as explored in reference [11] and [12].

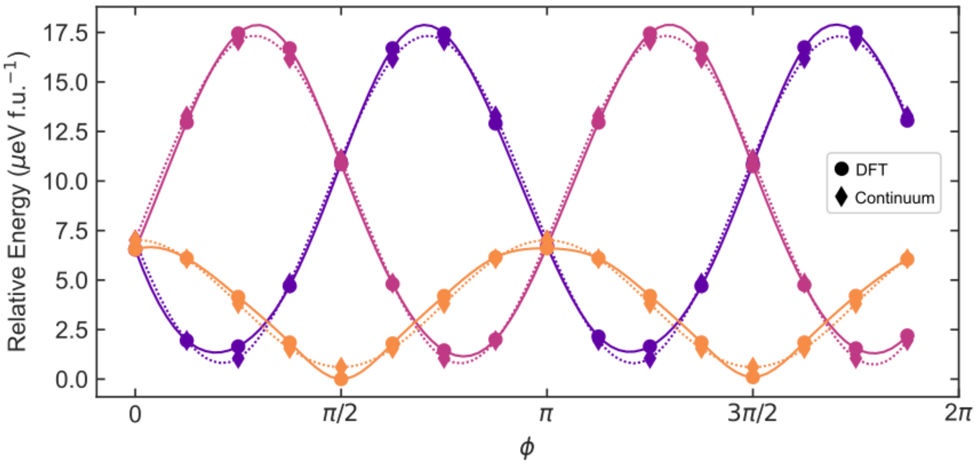

Supp. Figure S6 | Strain dependence of the cycloid propagation. Piecewise DFT calculations for the cycloid in the relaxed supercell, showing the dependence of $\boldsymbol{k}$. In the bulk crystal, the three possible cycloid directions are energetically equivalent, which makes sense given the 3-fold symmetry of the rhombohedral unit cell. In the epitaxially strained case, this 3-fold symmetry is disrupted by the biaxial strain in the (001) due to epitaxy, inducing a preferred direction within the (001). **c** Data in b overlayed with fits to **Eq. S1** shown as dashed lines.

Supp. Note S1 | Discussion of the magnetoelastic anisotropy.

From the calculations presented here, the effective anisotropy is greatly reduced for the strained unit cell. This can be reconciled from the insight of previous DFT studies, which computationally confirmed that the single ion anisotropy is most strongly affected by ferroelectric distortions [X], while the net DMI vector is most sensitive to the magnitude of antiferrodistortive rotations of oxygen octahedra [X]. From **Supp. Figure S5**, strain induces a dominant cooperative distortion of oxygen, relative to iron and bismuth ions, which reduces the magnitude of the effective anisotropy. We observe the quantitative outcome of these distortions in the reduction of $|K_{eff}|$ in **Supp. Table S1**. This helps to rationalize, if the strained cell is not allowed to relax individual atomic positions, the experimentally observed behavior for the cycloid $\boldsymbol{k}$ vector to orient along $[\bar{1}10]$, compared to $[\bar{1}01]$ and $[0\bar{1}1]$, is not reproduced.

In order to provide further evidence for the magnetoelastic anisotropy induced by epitaxial strain, the data here are also fit to the analytical expression for the energy versus rotation angle for BFO, derived in the SI of Ref. [13]. These results in **Supp. Figure S5** showcase an almost perfect agreement between the analytical expression and the data.

The anisotropy energy is defined in terms of the antiferromagnetic order parameter, oriented along the z-direction, which is parallel to [111]. K_{eff} includes both uniaxial magnetocrystalline anisotropy (MAE), as well as DMI energy contributions[13]. The magnetostrictive term accounts for plane strain in (001), where the unit vector defines the direction normal to the film plane with $\boldsymbol{P} \parallel [111]$ under no applied stress. This energy can be expressed as:

$$F' = F_{anis} + F_{MS} = -K_{eff}L_z^2 - U(\boldsymbol{L} \cdot \hat{n}) \qquad (S6)$$

where F' contains the effective anisotropy (F_{anis}) and plane-strain magnetostrictive (F_{MS}) contributions to the free energy and $\hat{n}$ is the unit vector that is normal to the thin film plane. Employing spherical coordinates, with the polar axis oriented along $\boldsymbol{P} \parallel [111]$ and the azimuthal axis along $[112]$, we can express the AFM order parameter as $\boldsymbol{L} = |\boldsymbol{L}|[\sin\theta\cos\phi, \sin\theta\sin\phi, \cos\theta]$. Both energy contributions can then be expressed in terms of the polar and azimuthal angles θ and ϕ:

$$F_{anis} = -K_{eff}\cos^2\theta \qquad (S7)$$

$$F_{MS} = -U\,(\sin\theta_n \sin\theta\cos\phi + \cos\theta_n\cos\theta)^2 \qquad (S8)$$

where θ_n defines the orientation of $\hat{n}$ within the polar reference frame. In the case of $\phi = \pi/2$, which corresponds to a $(11\bar{2})$ rotation plane,

$$F'_{11\bar{2}} = -\left(K_{eff} + U_{MS}\cos^2\theta_n\right)\cos^2\theta\,. \qquad (S9)$$

If $K_{eff} + U_{MS}\cos^2\theta_n < 0$, the $[111]$ axis is favored. If $K_{eff} + U_{MS}\cos^2\theta_n > 0$, however, intersections between the $(11\bar{2})$ and (111) planes (i.e. $[\bar{1}10]$ and $[1\bar{1}0]$) are preferred over the $[111]$ axis. This agrees with the energy versus rotation angle provided in **Figure 2**.

Supp. Table S1 | Fitted coefficients of Equation S1

	K_{eff} (μeV f.u.$^{-1}$)	U_{MS} (μeV f.u.$^{-1}$)	θ_n
Unstrained	19.655	-	-
Strained	-3.011×10^{-1}	-20.125	56.57°

In **Supp. Table S1**, we report the fitted coefficients of the energy terms in **Eq. S1** for both the unstrained, relaxed structure, as well as the unit cell constrained to the $DyScO_3$ lattice parameters, $a \cong b \cong 0.394$ nm. The uncertainties of these obtained values from scipy.optimize.curve_fit, are less than 10^{-14} eV for all K_{eff} and U_{MS} values, and 0.003° for θ_n. By comparison, for the unstrained structure, $\theta_n = 54.7°$ [5]. The coefficient of the magnetostrictive term is negative, $U_{MS} < 0$, which is expected under compressive epitaxial strain, based on the intuition provided by Ref. [13]. According to the calculation presented here, $|U_{MS}| > |K_{eff}|$, which further supports the claim that in $BiFeO_3$, strain strongly affects the easy axis/plane preference and orientation.

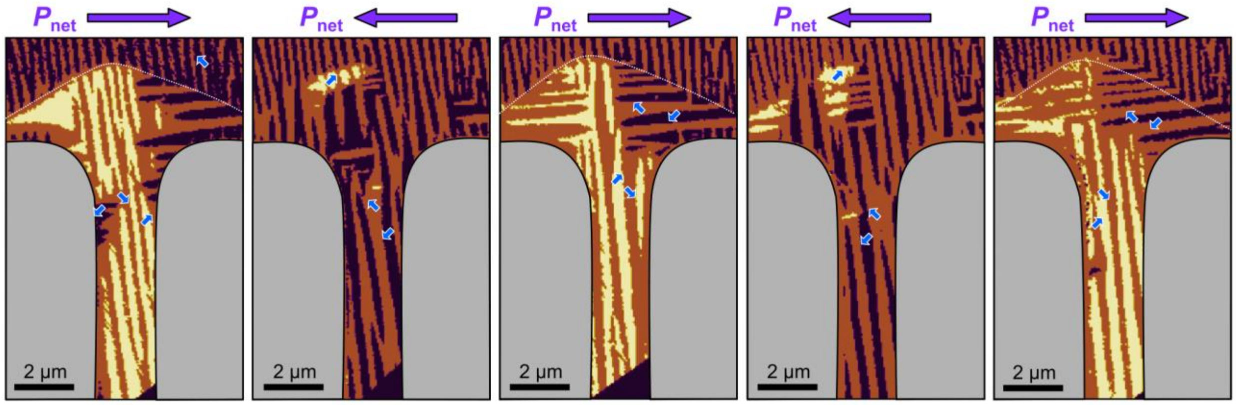

Supp. Figure S7 | Ferroelectric domain switching with applied electric field. Normalized PFM phase maps showing the reorientation of ferroelectric domains under successive electric fields. While ferroelectric domain walls remain generally stationary, with individual domains undergoing 71° in-plane switching, they are not completely so and may move with larger electric fields and longer pulse times.

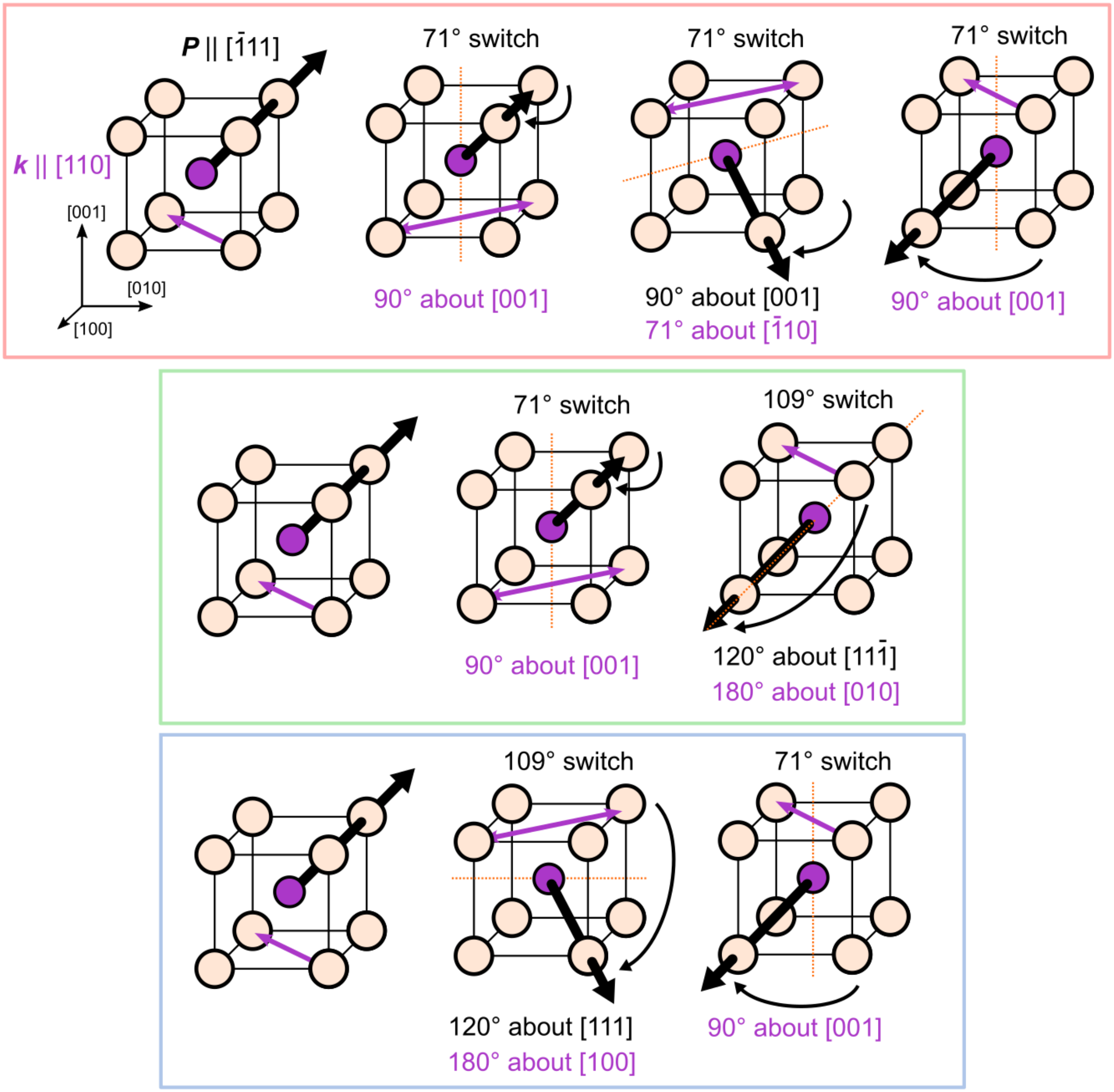

Supp. Figure S8 | Switching pathways of *P* and *k*. Shown previously in BFO, in 180° ferroelectric switching events *P* will rotate continuously through a combination of in-plane-71° and 109° switches. These three possible pathways with *P* starting along [$\bar{1}$11] are shown here. Operations shown in black are the higher symmetry operations that rotate *P*, but purple operations must be taken to preserve the experimentally observed sense of *k*. Because of the anisotropy in *k*, not all switching events can occur (in pseudocubic notation) through the highest

symmetry operations, for example C_3 rotations through the high symmetry $\langle \mathbf{111} \rangle$ axes, they must proceed through lower symmetry operations to preserve the in-plane nature of $\boldsymbol{k}$.

References

1. Gross, I. *et al.* Real-space imaging of non-collinear antiferromagnetic order with a single-spin magnetometer. *Nature* **549**, 252 (2017).
2. Zhong, H. *et al.* Quantitative Imaging of Exotic Antiferromagnetic Spin Cycloids in BiFeO3 Thin Films. *Phys. Rev. Appl.* **17**, 044051 (2022).
3. Chen, Z. H., Damodaran, A. R., Xu, R., Lee, S. & Martin, L. W. Effect of “symmetry mismatch” on the domain structure of rhombohedral BiFeO3 thin films. *Applied Physics Letters* **104**, 182908 (2014).
4. Munro, J. M. *et al.* Discovering minimum energy pathways via distortion symmetry groups. *Phys. Rev. B* **98**, 085107 (2018).
5. Ederer, C. & Spaldin, N. A. Weak ferromagnetism and magnetoelectric coupling in bismuth ferrite. *Physical Review B - Condensed Matter and Materials Physics* **71**, 1–4 (2005).
6. Liechtenstein, A. I., Anisimov, V. I. & Zaanen, J. Density-functional theory and strong-interactions - orbital ordering in mott-hubbard insulators. (1995).
7. Dudarev, S. L., Botton, G. A., Savrasov, S. Y., Humphreys, C. J. & Sutton, A. P. Electron-energy-loss spectra and the structural stability of nickel oxide: An LSDA+U study. *Phys. Rev. B* **57**, 1505–1509 (1998).
8. Mellan, T. A., Corà, F., Grau-Crespo, R. & Ismail-Beigi, S. Importance of anisotropic Coulomb interaction in LaMnO3. *Phys. Rev. B* **92**, 085151 (2015).
9. Streltsov, S. V. & Khomskii, D. I. Orbital physics in transition metal compounds: new trends. *Phys.-Usp.* **60**, 1121 (2017).
10. Pavarini, E. *Correlated Electrons: From Models to Materials: Lecture Notes of the Autumn School Correlated Electrons 2012 at Forschungszentrum Jülich, 3 - 7 September 2012*. (Forschungszentrum Jülich, Zentralbibliothek, Verlag, 2012).
11. Streltsov, S. V. & Khomskii, D. I. Jahn-Teller Effect and Spin-Orbit Coupling: Friends or Foes? *Phys. Rev. X* **10**, 031043 (2020).
12. Poluyanov, L. V. & Domcke, W. Jahn-Teller, pseudo-Jahn-Teller, and spin-orbit coupling Hamiltonian of a d electron in an octahedral environment. *The Journal of Chemical Physics* **137**, 114101 (2012).
13. Sando, D. *et al.* Crafting the magnonic and spintronic response of $BiFeO_3$ films by epitaxial strain. *Nature Materials* **12**, 641–646 (2013).

14. Dovzhenko, Y. *et al.* Magnetostatic twists in room-temperature skyrmions explored by nitrogen-vacancy center spin texture reconstruction. *Nat Commun* **9**, 2712 (2018).
